# Audit of availability and distribution of paediatric cardiology services and facilities in Nigeria

**DOI:** 10.5830/CVJA-2016-057

**Published:** 2017

**Authors:** Ekanem N Ekure, Wilson E Sadoh, Fidelia Bode-Thomas, Christopher S Yilgwan, Adeola A Orogade, Adeola B Animasahun, Oluwatoyin O Ogunkunle, Samuel I Omokhodion, Iretiola Babaniyi, Maxwell U Anah, Barbara E Otaigbe, Adebiyi Olowu, Frances Okpokowuruk, Ogechi C Maduka, Uvie U Onakpoya, Daberechi K Adiele, Usman. M Sani, Mustapha Asani, Queennette Daniels, Chinyere C Uzodimma, Chika O Duru, Mohammad B Abdulkadir, Joseph K Afolabi, John A Okeniyi

**Affiliations:** Department of Paediatrics, College of Medicine, University of Lagos, Lagos, Nigeria; Paediatric Cardiology Unit, Department of Child Health, University of Benin Teaching Hospital, Benin City, Nigeria; Department of Paediatrics, University of Jos, Nigeria; Department of Paediatrics, University of Jos, Nigeria; Department of Paediatrics, Ahmadu Bello University Teaching Hospital, Zaria, Nigeria; Department of Paediatrics and Child Health, Lagos State University College of Medicine, Ikeja, Lagos, Nigeria; Division of Paediatric Cardiology, Department of Paediatrics, University College Hospital, Ibadan, Nigeria; Division of Paediatric Cardiology, Department of Paediatrics, University College Hospital, Ibadan, Nigeria; Department of Paediatrics, National Hospital, Abuja, Nigeria; Department of Paediatrics, University of Calabar Teaching Hospital, Calabar, Nigeria; Department of Paediatrics, College of Health Sciences, University of Port Harcourt, Nigeria; Department of Paediatrics, Olabisi Onabanjo University Teaching Hospital, Sagamu, Nigeria; Department of Paediatrics, University of Uyo Teaching Hospital, Uyo, Nigeria; Department of Paediatrics, Federal Staff Hospital, Abuja, Nigeria; Surgery Unit, Bikets Medical Centre, Osogbo, Nigeria; Department of Paediatrics, University of Nigeria Teaching Hospital, Enugu, Nigeria; Department of Paediatrics, Usmanu Danfodiyo Teaching Hosptial, Sokoto, Nigeria; Department of Paediatrics, Aminu Kano Teaching Hospital, Kano City, Nigeria; Paediatrics Unit, Zankli Hospital, Abuja, Nigeria; Department of Paediatrics, Federal Medical Centres, Abeokuta, Nigeria; Department of Paediatrics and Child Health, Niger Delta University Teaching Hospital, Wilberforce Island, Bayelsa State, Nigeria; Department of Paediatrics and Child Health, University of Ilorin, Ilorin, Nigeria; Department of Paediatrics and Child Health, University of Ilorin, Ilorin, Nigeria; Department of Paediatrics, College of Health Sciences, Obafemi Awolowo University, Ile-Ife, Nigeria

**Keywords:** audit, Nigeria, paediatric cardiac services, open-heart surgery

## Abstract

**Background:**

Paediatric cardiac services in Nigeria have been perceived to be inadequate but no formal documentation of availability and distribution of facilities and services has been done. Objective: To evaluate and document the currently available paediatric cardiac services in Nigeria.

**Methods:**

In this questionnaire-based, cross-sectional descriptive study, an audit was undertaken from January 2010 to December 2014, of the personnel and infrastructure, with their distributions according to geopolitical zones of Nigeria.

**Results:**

Forty-eight centres participated in the study, with 33 paediatric cardiologists and 31 cardiac surgeons. Echocardiography, electrocardiography and pulse oximetry were available in 45 (93.8%) centres while paediatric intensive care units were in 23 (47.9%). Open-heart surgery was performed in six (12.5%) centres. South-West zone had the majority of centres (20; 41.7%).

**Conclusions:**

Available paediatric cardiac services in Nigeria are grossly inadequate and poorly distributed. Efforts should be intensified to upgrade existing facilities, establish new and functional centres, and train personnel.

## Background

An estimated 70 000 children are born with congenital heart disease (CHD) in Nigeria annually, based on seven million annual births and a global CHD incidence of 1%.^1^,^2^ There are an estimated 300 000 school-aged children afflicted with rheumatic heart disease (RHD).^3^ The prevalence of RHD in a Nigerian community was 57/100 000 school children.^4^ The burdens of other acquired heart diseases (AHD) of childhood, although perceived to be significant, have not been quantified. In addition, most of the systemic diseases of childhood, especially infections and metabolic conditions, affect the heart in one way or another. All these constitute a huge demand for paediatric cardiac services in the country.

The poor availability of paediatric cardiac services in developing countries is well documented.^5^ Provision of paediatric cardiac services and uneven distribution of such services in the most populous sub-Saharan African country presents a huge challenge. Currently, most of the surgical paediatric cardiac needs of Nigerian children are being met outside the country, with only a small number of affected children receiving their interventions in the country during periodic medical missions undertaken by specialists from within and outside Nigeria.^6^,^7^ It is therefore generally perceived that the number of paediatric cardiac practitioners and the facilities for paediatric cardiac care in Nigeria are grossly insufficient to meet the huge demand for such services.

Although the last half decade has witnessed efforts to train personnel, establish new centres or upgrade the capacities of old centres, there has been no previous attempt to formally document the human and infrastructural resources available to provide paediatric cardiac services in Nigeria. We therefore set out to document the currently available services and resources as a baseline for future comparison, with the hope that the needs gap will be brought more sharply into focus and serve to spur more vigorous attempts at bridging the gap. It will also help improve access to care and ease referral decisions by informing practitioners on the available services that are closest to their patients.

## Methods

The Federal Republic of Nigeria is the most populous African nation, with an estimated population of more than 177 million people.^8^ There are 36 states and the Federal Capital Territory, which are grouped into six geopolitical zones. Each state has at least one government-owned designated tertiary health centre but not all of them have the capacity to investigate and definitively diagnose paediatric cardiac conditions. Fewer still have the capacity to undertake open-heart surgeries and other cardiac interventions. A few private medical centres however, have relatively advanced capabilities for paediatric cardiovascular diagnostic and interventional services.

A structured questionnaire was sent to all Federal Government-owned tertiary health facilities and to large private medical centres in Nigeria in February 2015. The centres known to have a paediatric cardiologist on staff were included. This was done mainly through the platform of the Nigerian Paediatric Cardiologists’ network, an internet platform for disseminating information among paediatric cardiologists, interested paediatricians and paediatric cardiac surgeons in Nigeria. The questionnaire items included the number and type of personnel and the range of paediatric cardiac facilities and services available in each centre in the period between January 2010 and December 2014. Only one questionnaire was to be returned per centre. Ethical approval was not required to use data from the audit.

## Statistical analysis

The information provided was coded and entered into SPSS version 20.0 (Chicago, Illinois). The proportions of centres with particular cardiac services were expressed as percentages. Available personnel and infrastructure were analysed according to geopolitical zone.

## Results

**Fig. 1.**
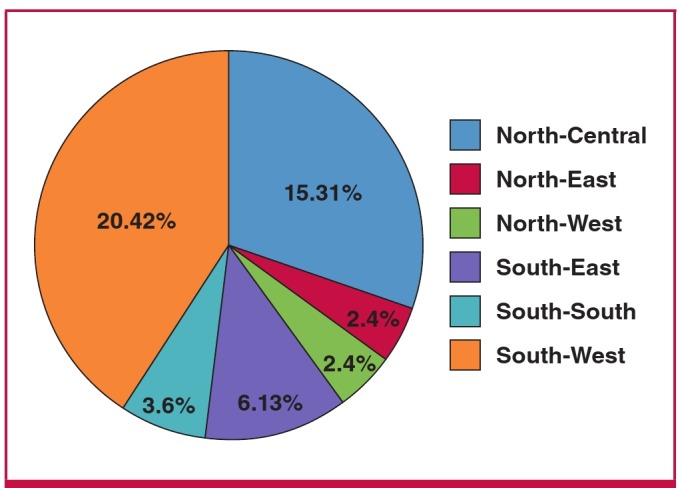
Distribution of the participating centres according to geopolitical zone.

There was a 100% response rate from the 48 centres that participated in the study, of which 25 (52.1%) were government owned. The majority of the participating centres (20; 41.7%) were located in the South-West geopolitical zone. The distribution of centres in the other zones is shown in Fig. 1.

A total of 33 paediatric cardiologists were practicing full time in 26 of the 48 centres, providing cardiac services to the 87 million population of children in Nigeria.^1^ The remaining 22 centres had only visiting cardiologists. All 33 physicians were able to perform echocardiography. A total of 31 cardiac surgeons were identified in this survey. In 12 centres, there were 19 (61.3%) surgeons trained in adult cardiac surgery only, while 10 centres had 12 (38.7%) surgeons trained in both adult and paediatric cardiac surgery. The distribution of the other cadres of personnel is shown in Table 1. Table 2 depicts the distribution of personnel according to geopolitical zone

**Table 1:** Distribution of paediatric cardiac personnel in the centres

**	**	*Total number of personnel in 48 centres*		
*Personnel type*	*No of centres*	*Total*	*Median*	*Range*
Physicians with echo capability	26	33	1	1–4
Adult surgeons	21	31	1	1–6
Paediatric surgeons	10	12	1	1–2
Interventionists	7	5	1	1–1
Cardiac nurses	14	97	1.5	1–23
Cardiac anaesthetists	14	22	1	1–3
Perfusionists	9	13	1	1–2
Intensivists	5	7	1	1–2

Some personnel (physicians, surgeons, anaesthetiscs, perfusionists, nurses and interventionists) were working in more than one centre. Some adult surgeons also functioned as paediatric surgeons.

**Table 2:** Distribution of personnel by geopolitical zone

**	*NC*	*NE*	*NW*	*SE*	*SS*	*SW*	**
*Type of personnel*	*n (%)*	*n (%)*	*n (%)*	*n (%)*	*n (%)*	*n (%)*	*Total*
Physicians that can perform echo	11 (33.3)	0 (0.0)	4 (12.1)	3 (9.1)	6 (18.2)	9 (27.3)	33
Adult surgeons	7 (22.6)	0 (0.0)	3 (9.7)	7 (22.6)	6 (19.4)	8 (25.8)	31
Paediatric surgeons	1 (8.3)	0 (0.0)	2 (16.7)	2 (16.7)	1 (8.3)	6 (50.0)	12
Interventionists	1 (20.0)	0 (0.0)	0 (0.0)	0 (0.0)	1 (20.0)	3 (60.0)	5
Nurses	11 (11.6)	0 (0.0)	4 (4.2)	25 (26.3)	8 (8.4)	47 (49.5)	95
Anaesthetists	2 (11.1)	0 (0.0)	2 (11.1)	2 (11.1)	3 (16.7)	10 (55.6)	18
Perfusionist	1 (7.7)	0 (0.0)	1 (7.7)	2 (15.4)	1 (7.7)	8 (61.5)	13

NC = North-Central, NE = North-East, NW = North-West, SE = South-East, SS = South-South and SW = South-West.

Forty-seven (97.9%) centres had equipment for electrocardiography (ECG) and pulse oximetry, while echocardiography could be performed in 45 (93.8%) centres. In the three centres without an echocardiography machine, the facility had previously been available but was not functioning at the time of the survey. The centres with functional echocardiography machines had facilities for paediatric probe with transducer frequency of at least 5 MHz, two-dimensional, colour and Doppler facilities. Although defibrillators were available in 23 (47.9%) centres, paediatric paddles were only available in 11 (47.8%) of these. The distribution of the other equipment according to geopolitical zone is shown in Table 3.

**Table 3:** Availability of equipment in centres according to geopolitical zone

**	*NC*	*NE*	*NW*	*SE*	*SS*	*SW*	**
*Type of equipment*	*n (%)*	*n (%)*	*n (%)*	*n (%)*	*n (%)*	*n (%)*	*Total*
Echo machines	15 (33.3)	2 (4.4)	2 (4.4)	3 (6.7)	4 (8.9)	19 (42.2)	45
ECG	15 (31.9)	2 (4.3)	2 (4.3)	3 (6.4)	6 (12.8)	19 (40.4)	47
Holter monitor	7 (33.3)	1 (4.8)	0 (0.0)	2 (9.5)	2 (9.5)	9 (42.9)	21
Defibrillator	7 (29.2)	1 (4.2)	1 (4.2)	2 (8.3)	4 (16.7)	9 (37.5)	24
Catheterisation lab	1 (16.7)	0 (0.0)	1 (16.7)	1 (16.7)	0 (0.0)	3 (50.0)	6
ICU	6 (24.0)	2 (8.0)	2 (8.0)	2 (8.0)	4 (16.0)	9 (36.0)	25
Ventilators	10 (21.7)	0 (0.0)	1 (2.2)	17 (40.0)	4 (8.7)	14 (30.4)	46

Echo = echocardiography, ICU = intensive care unit, ECG = electrocardiogram.

NC = North-Central, NE = North-East, NW = North-West, SE = South-East, SS = South-South and SW = South-West.

Cardiac catheterisation was available in six (12.5%) of the 48 centres but was utilised for children in only three centres (6.3%) for diagnostic and therapeutic purposes. All six centres had single-plane equipment. The therapeutic indications included percutaneous closure of patent ductus arteriosus (PDA) and atrial septal defects (ASD). Surgical operations were performed for congenital heart diseases in 13 centres.

There were 84 cardiac surgeries for congenital heart diseases performed in 13 centres with a median (range) of six (1–20) operations per centre over the study period. The average annual number of surgeries for congenital heart disease was 21. The surgeries included ligation of PDA 51 (60.7%), total repair of tetralogy of Fallot (TOF) 16 (19.0%), ASD eight (9.5%), ventricular septal defect (VSD) seven (8.3%), and Blalock- Taussig shunt (B-T shunt) two (2.4%) for TOF and double-outlet right ventricle (DORV). There were a total of 21 rheumatic surgical cases performed in four centres during the study period. Of the 13 centres, only six (46.2%) had facilities for and performed open-heart surgery on children. The others did mostly PDA ligation. Pacemakers were inserted in seven (14.6%) centres.

**Fig. 2.**
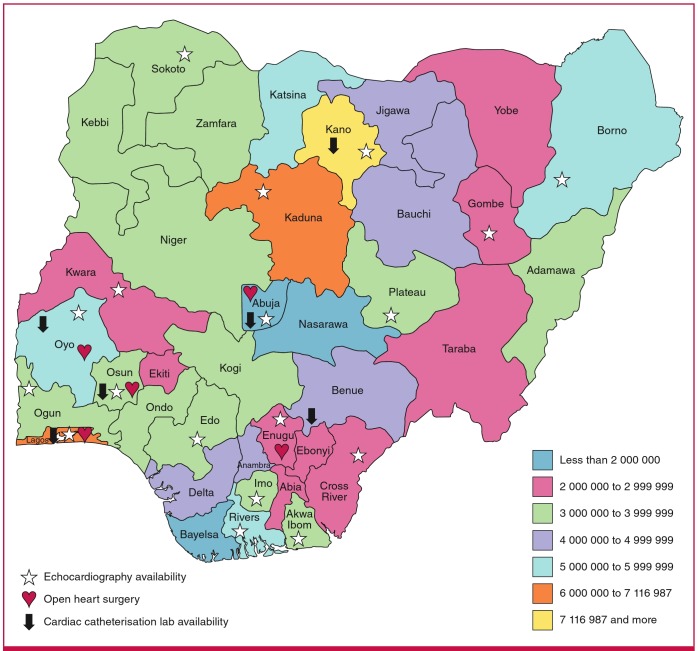
Population map of states with echocardiography, cardiac catheterisation and open-heart surgery facilities.

Of the six centres that had performed open-heart surgery on children, four (66.7%) were located in the South-West geopolitical zone, which also housed three (50%) of the six centres with catheterisation laboratories. The centres with open-heart surgery and cardiac catheterisation facilities and their distribution according to geopolitical zone are shown in Table 4, while the availability of echocardiography, cardiac catheterisation and open-heart surgery by state in the country is depicted in Fig. 2.

**Table 4:** Centres with open-heart surgery and cardiac catheterisation facilities by geopolitical zone

*Centre according to zone*	*Open-heart surgery*	*Catheterisation lab*
South-West zone		
Lagos University Teaching Hospital, Lagos	✓	
Lagos State University Teaching Hospital, Lagos	✓	
Redington Hospital, Lagos		✓
University College Hospital, Ibadan	✓	✓
Biket Medical Centre, Osogbo	✓	✓
North-Central zone		
Heart scan, Abuja		✓
Garki Hospital, Abuja	✓	
South-East zone		
University of Nigeria Teaching Hospital, Enugu*	✓	✓
North-West zone		
Aminu Kano Teaching Hospital, Kano*		✓

*Cardiac catheterisation for children is yet to commence in these centres.

Twenty-five (52.1%) of the 48 participating centres had intensive care units (ICUs), with a total of 140 ICU beds between them [median (range): 5.5 (2– 6) beds]. Only 10 (40.0%) of the 25 centres however had a dedicated paediatric ICU with 24 beds. The median (range) number of beds in the paediatric ICU was one (1–5). Seventeen (35.4%) of the 48 centres had a total of 46 ventilators [median (range): one (1–7) ventilators].

The distribution of centres with open-heart surgery facilities, catheterisation laboratories, ICU and ventilators according to the type of hospital is shown in Table 5. All 23 private centres except one were manned by visiting cardiologists from other centres, while in three (50%) centres, the surgeons were visiting.

**Table 5:** Distribution of number of centres with some facilities and procedures according to the type of hospital

**	*Public*	*Private*	*Total*
*Facilities/procedures*	*n (%)*	*n (%)*	*n (%)*
Open-heart surgery	4 (66.7)	2 (33.3)	6 (100.0)
Catheterisation lab	3 (50.0)	3 (50.0)	6 (100.0)
Intensive care unit	21 (84.0)	4 (16.0)	25 (100.0)
Ventilators	41 (89.1)	5 (10.9)	46 (100.0)

## Discussion

The study found a total of 33 paediatric cardiologists serving the country of 87.7 million children, or one paediatric cardiologists to 2.6 million children. This falls far short of the ideal requirement of a minimum of one paediatric cardiologist to serve 0.5 million of the population.^9^ Therefore Nigeria would need a minimum of 174 paediatric cardiologists to attend to her 87.7 million children. The available 33 paediatric cardiologists, which is about one-fifth (33/174) of the number of personnel actually required, speaks to the monumental challenge before Nigeria in providing adequate paediatric cardiac services.9 The proportion is smaller than the 24/88 (27%) reported paediatric cardiologists serving a population of 44 million in South Africa, or one to 1.8 million people, which is also considered inadequate.^10^

The number of surgeons recorded in the present study is also far lower than the recommended number of at least two surgeons per centre. The reasons for the lower proportion of paediatric cardiologists per total number of children in Nigeria include poor training facilities in the country, such that residents intending to do paediatric cardiology or cardiothoracic surgery have part or all of their training in centres outside the country. Such residents may be unwilling to return to the country to pursue a career with the prospect of poor or lack of equipment and materials to work with. Furthermore, not many doctors may be interested in pursuing a career in cardiology and cardiac surgery because of the long duration of training and the ill-equipped training facilities.

The deficiency of manpower is further underscored by the fact that some of these few available personnel were also visiting physicians and surgeons to other centres, particularly private ones. This not only highlights the need to train more personnel, but in the opinion of the study group, also could point to inability of the private centres to provide adequate remuneration, tenured appointments and job security. This is against the norm in other countries where private hospital services are able to attract personnel from state-owned hospitals.

This is not surprising, given the capital-intensive nature of cardiac surgery, coupled with its non-inclusion in the National Health Insurance Scheme.11 The private centres are therefore unable to generate enough income to pay highly skilled full-time staff, especially as they must rely on intermittent surgical missions to be able to generate adequate numbers of paying patients on whom to operate. These highly skilled staff therefore remain in government employment, providing low levels of cardiologyservice but willing to offer their services to those private centres that are also beginning to develop cardiac programmes whenever they organise such missions.

This state of affairs leaves a lot to be desired, because practitioners require a minimum number of procedures to maintain competence and may need to periodically visit established centres outside the country to satisfy this requirement. This does not augur well for the rapid development of paediatric cardiac services in the country and creates some kind of expensive but inefficient vicious circle. Urgent intervention in terms of massive investment in the health sector and new ways of healthcare financing will be required to break the vicious circle. However, with many other competing needs in and outside the health sector, this slow pace of development of paediatric cardiac services may continue for some time.

The North-Eastern geopolitical zone is particularly badly affected, with a severe dearth of paediatric cardiology services and personnel, despite having 14% of the population of Nigera.12 This may be due to the fact that it is the zone that has been particularly hit by insurgency activities in recent years. This means that parents of affected children will have to defy many odds to obtain the necessary care for their children.

The other two northern zones (North-West and North-Central) also lag behind the south, especially the South-West, in terms of paediatric cardiac infrastructure. This finding is in tandem with a report of the paediatrician work force in Nigeria, where more than two-thirds of paediatricians practice in the South, with the lowest child:paediatrician ratio in the South- West.13 This trend is also consistent with observations in other aspects of socio-economic development, with the South-West historically leading the pace in terms of Western education and its attendant developments.

While detailed echocardiography is adequate to prepare most patients for surgery, cardiac catheterisation is needed for the anatomical and physiological assessment of patients with CHD on whom echocardiographic evaluation is difficult.14 It is also being increasingly used for minor interventions that were hitherto surgically repaired. Only three out of the six available catheterisation laboratories in the country provided services for children during the study period, although more recently, three additional catheterisation laboratories were either in installation phase or were awaiting official opening of the centres. This again highlights inadequacy of equipment for a large population.

The rudimentary paediatric cardiology and cardiac surgery services in Nigeria are being provided mainly by governmentowned centres, no doubt because of the huge capital outlays involved in setting one up. The government centres however have their peculiar challenges, such as incessant industrial action, frequent power interruption, unnecessary bureaucracy and intra-institutional conflict, resulting in loss of public confidence. One way of strengthening these centres might be regional co-operation between centres by effective referrals to one or two centres that perform open-heart surgery, considering the small number of surgeries performed in the country. As the number of surgeries outstrip the capacity of existing centre(s) to peform open-heart surgery, other centres with adequate facilities can be upgraded to referral centres. The sharing of facilities and expertise ensures sustainability of the few available centres.

The small number of surgeries performed locally is the underlying reason for the thriving medical tourism for openheart surgery outside Nigeria. The quest to seek medical care, including paediatric cardiac care outside Nigeria where more comprehensive cardiac services are readily available has not only led to a lot of capital flight but has also provided little opportunity for development of the fledgling cardiac services in the country. It is estimated that Nigerians spend about $20 billion on health costs annually outside Nigeria.^15^ The solution is the provision of cardiac services at standards close to or on par with those outside the country to convince the populace to patronise the services available in the country.

The cost of cardiac care is not cheap anywhere in the world. In a single-centre study in Nigeria, the cost of open-heart surgery was found to range between US$6 230 and US$11 200 in a country where the GNI per capita income is US$2 760.^16^ A previous study demonstrated the catastrophic health cost to families who pay out of pocket for their children’s medical care.^17^ Although the cost of cardiac surgeries/interventions in Nigeria is cheaper than in many centres internationally, most Nigerian families cannot afford it. In a bid to bridge this gap, some non-governmental organisations, such as the Kanu Heart Foundation, Save a Child’s Heart Nigeria, and a number of other faith-based and non-faith-based organisations have provided full funding or have subsidised the cost of surgeries abroad for a small number of affected children.

## Conclusion

The available paediatric cardiac services in Nigeria are grossly inadequate and poorly distributed to cater for the teeming population. The use of periodic medical missions to accomplish intervention in a few selected cases, while marginally reducing the burden of children with uncorrected cardiac anomalies, will only serve a short-term remediation of the problem. Medium- and long-term approaches would be the upgrading of existing centres, strengthening of referral systems, coupled with the training and re-training of relevant personnel to man the centres. There is a need for better public–private partnership. It is important that efforts by government and non-governmental organisations in providing funding for surgeries abroad be continued until the cardiac services in the country are adequate for the needs of Nigerian children with structural cardiac defects.
